# An influence of family history and season of birth on clinical characteristics of schizophrenia

**DOI:** 10.1192/j.eurpsy.2023.1054

**Published:** 2023-07-19

**Authors:** T. Lezheiko, V. Plakunova, N. Kolesina, V. Mikhailova, V. Golimbet

**Affiliations:** Clinical Genetics Laboratory, Mental Health Research Center, Moscow, Russian Federation

## Abstract

**Introduction:**

A large body of evidence shows that both genetic and environmental factors play an important role in the etiology of schizophrenia. There is also growing evidence of an interaction between these factors.

**Objectives:**

To investigate the influence of family history of schizophrenia, which reflects the contribution of genetic factors, and birth in the winter months, considered as an important environmental risk factor for this disease, on the clinical characteristics of schizophrenia.

**Methods:**

The results of a clinical examination of 1590 inpatients with schizophrenia (F20.0 ICD-10) were analyzed. The analysis included clinical characteristics of the disease (age of onset of the disease, severity of symptoms, which were assessed by PANSS), information about the family history of schizophrenia, and the season of birth (SOB) of the patient. Data analysis was carried out using multivariate analysis of variance, in which clinical characteristics were used as a dependent variable, and the presence/absence of schizophrenia in first-degree relatives of the patient, patient’s birth in the winter months/birth in other months of the year, sex were independent factors.

**Results:**

The study group included 1153 women and 437 men. Family history was present in 569 patients (427 women) and absent in 1021 (726 women). In 415 (26.1%) cases, patients born in the winter months. In the group without family history, the percentage of births in the winter months did not differ from that in the group with family history. There was an effect of sex on the age of onset of the disease as well as on the severity of positive symptoms, which were higher in women (p =0.0000). Family history of schizophrenia had a significant, sex-independent effect on the age of onset. The disease manifested at an earlier age in patients with family history. Being born in the winter months was not associated with either age of onset or positive symptoms. There were significant main effects of family history (p=0.018) and birth in the winter months (p=0.044) on negative and general psychopathological symptoms, which were not mediated by sex. The additive effect of these factors was found at the trend level (p=0.08), while the greatest severity of negative symptoms was observed in the group with a family history and birth in the winter months (p=0.02). A significant additive effect (p=0.012) of family history and birth in the winter months on general psychopathological symptoms was found. As in the case of negative symptoms, the greatest severity was noted in the presence of both factors.

**Conclusions:**

Family history and birth in the winter months are not only risk factors for the development of schizophrenia, but also affect the severity of the disease. The results indicate the feasibility of further molecular genetic studies on this sample, both using candidate gene analysis and genome-wide analysis with polygenic risk assessment.

**Disclosure of Interest:**

None Declared

